# The value of prostate cancer support groups: a pilot study of primary physicians’ perspectives

**DOI:** 10.1186/1471-2296-15-56

**Published:** 2014-03-28

**Authors:** Bernard M Garrett, John L Oliffe, Joan L Bottorff, Michael McKenzie, Christina S Han, John S Ogrodniczuk

**Affiliations:** 1 School of Nursing, University of British Columbia, T201, 2211 Wesbrook Mall, V6T 2B5 Vancouver, BC, Canada; 2 School of Nursing, University of British Columbia Okanagan, FIP246A - Fipke Centre, 3333 University Way, V1V 1 V7 Kelowna, BC, Canada; 3 British Columbia Cancer Agency, Vancouver Centre, 600 West 10th Avenue, V5Z 4E6 Vancouver, BC, Canada; 4 Department of Psychiatry, University of British Columbia, 420 - 5950 University Blvd., V6T 1Z3 Vancouver, BC, Canada

**Keywords:** Prostate cancer support groups, Primary healthcare providers, Clinician’s attitudes

## Abstract

**Background:**

In Canada, prostate cancer (PCa) is the most common male cancer, and prostate cancer support groups (PCSGs) have prevailed for more than 20 years providing support to men with PCa and their families. While the format, focus and benefits of attending PCSGs have been reported little is known about primary physicians’ (PPs) perceptions of these groups. This article describes Canadian primary physicians’ views about face-to-face and web-based PCSGs.

**Methods:**

Canadian based primary physicians (n = 140) attending a 2012 Continuing Medical Education Conference participated in a pilot survey questionnaire study. The 56-item questionnaire used in this study included six sets of attitudinal items to measure primary physicians’ beliefs about positive and negative influences of PCSGs, reasons for attending PCSGs, the attributes of effective PCSGs, and the value of face-to-face and web-based PCSGs.

**Results:**

Results showed that PCSGs were positively valued, particularly for information sharing, education and psychosocial support. Poor inclusivity, privacy, and accessibility were identified as potential barriers, and recommendations were made for better marketing and web-based PCSGs to increase engagement with potential attendees.

**Conclusions:**

Findings suggest PPs highly valued the role and potential benefits of PCSGs. Information provision and an educational role were perceived as key benefits amid the need to improve local and provincial marketing of PCSGs. The potential for web-based PCSGs to help in the support of PCa patients was also recognized.

## Background

In Canada, prostate cancer (PCa) is the most common male cancer [1] with widespread availability of prostate specific antigen (PSA) testing increasing PCa detection [2,3]. Men are also living longer with PCa and amid declining PCa mortality rates there are expectations that the PCa incidence will increase significantly in the future [1,4]. Today many men experience PCa as a chronic illness [5], for which ongoing psychosocial supports are needed. Among the support options available, volunteer-led, community-based face-to-face prostate cancer support groups (PCSGs) have prevailed as a major source of such support in Canada for more than 20 years, attracting men and their partners to monthly meetings at approximately 100 Canadian groups. Another recent trend is Web-based PCSGs [6].

Research findings indicate that attending PCSGs provides reassurance, reduces anxiety, improves positive outlook and the perception of being more involved in treatment decisions [7,8]. Other reported benefits include mitigating the psychosocial impact of cancer by conveying information, empowering men with PCa, enhancing and facilitating psychosocial adjustment, and helping men and their partners cope with PCa [8-19]. A 2005 review [20] concluded that PCSGs attendees most valued the information and education they received from attending group meetings (e.g., information related to treatment, side effects, and the latest PCa research). Reported barriers to attending PCSGs included men’s tendencies to avoid disclosure (due to a low perceived need for support), fear of stigmatization, denial of illness, practical access issues, the desire to avoid burdening others, [21-23] and misperceptions that PCSG meetings were geared towards support of the terminally ill [24].

While PCSG attendees and non-attendees perceptions have been described, little is known about primary physicians’ (PPs) perceptions of PCSGs (both face-to-face and Web based). PPs’ views about PCSGs are especially important because there is evidence that health care providers (HCPs) strongly influence men’s interest in attending PCSGs [25-27]. Indeed research confirms that HCPs’ endorsement is a leading influence of patients’ PCSG attendance [25,28,29]. Inversely, HCPs’ lack of awareness of PCSGs can reduce the likelihood of men attending PCSGs [23,27,30]. In a novel study of 36 Australian clinicians (27 urologists and 9 radiation oncologists), Steginga et al. [26] found participants were reluctant to refer patients to PCSGs, fearing that biased viewpoints and misinformation within the groups might contribute to men’s uncertainty and decisional regret. Whether these findings generalize to PPs more widely, or apply in the Canadian context is unknown.

In this study, our primary objective was to establish baseline data by addressing the question: what are PPs perceptions of the value of PCSGs?

## Methods

An initial pilot survey of PPs was undertaken, designed to test logistics and gather information prior to conducting a larger scale investigation. The pilot study was designed to collect baseline data from subjects and improve study design including consideration if the initial findings support the tools/approach selected, feasibility of the selected methods, and technical and cultural issues that should be addressed to improve the quality, rigour and efficiency of later work. In this inductive pilot study to elicit data on PPs perceptions regarding PCSGs a simple survey approach was adopted based on Steginga et al.’s tool [26]. This survey utilized both Likert scaled questions to gather quantitative data and open-ended questions to collect qualitative data to further explore the PPs perceptions.

### Subjects

Following review by the University of British Columbia’s Behavioural Research Ethics Board’s approval, a non-probability convenience sample of Canadian-based PPs was recruited in two ways. In the first week of launching the survey questionnaire (SQ) 14 subjects responded to advertisements and completed the survey online. Shortly thereafter, the study lead (second author) provided a brief background and the purpose of the study ahead of inviting approximately 1,400 CME delegates at the 2012 St. Paul’s Hospital Continuing Medical Education (CME) conference, Vancouver, British Columbia to complete the SQ. Two research staff distributed hard copy SQs and postcard flyers with details for accessing the SQ online at the CME conference and 126 attendees completed the survey in hard copy (n = 93) and online (n = 33). All participants were offered an honorarium of a $50 gift card as an incentive acknowledging their contribution to the study. For the pilot study there were no specific exclusion criteria and this group was selected as it represented a diverse range of PPs from a variety of provinces and urban and rural areas who could be expediently accessed to provide some initial data.

### Survey instrument

The instrument selected to solicit the PP’s views was composed of a 56-item questionnaire based on an existing Australian study tool [26]. This tool, was developed from findings drawn from qualitative interviews with Australian-based HCPs, and subsequently pilot-tested and validated with 36 PCa specialists in 2006. We incorporated five demographic questions and six sets of attitudinal items to measure beliefs about: positive influences of PCSGs; negative influences of PCSGs; reasons for attending PCSGs; the attributes of effective PCSGs; and the value of face-to-face and web-based PCSGs. Each set of attitudinal items included 5–9 questions that were each rated by respondents using a 5-point Likert scale (where 1 = strongly disagree and 5 = strong agree). An open-ended question to provide additional information was also included in the survey: “Are there any other comments you would like to make about prostate cancer support groups and/or this survey?” This instrument is available from the researchers on request. Descriptive univariate statistics were used to analyze the survey responses using median scores (being more appropriate for ordinal Likert data). An interpretive content analysis of responses to the open-ended questions was also undertaken and the participant’s responses were read, coded and analyzed to identify and report key thematic elements and patterns emerging from the data.

## Results

September 2012 through January 2013, a total of 140 Canadian PPs in a variety of settings including hospital and/or private practice completed the survey in hard copy (n = 93: 66%) and online (n = 47: 34%). Women comprised 61% (n = 85), and respondents ranged in age from 27 to 69 years (Median = 44.1 years). The majority (55%) had over 5 years’ experience with PCa patients, and 11% had over 30 years of experience with this patient population. The remaining 45% of respondents indicated that they had less than five years’ experience with this patient group. A minority of PPs in the sample (36%) reported actually referring patients to PCSGs, and few (6%) reported presenting content at PCSGs. For more respondent demographic details, please refer to Table 1. Demographics.

**Table 1:** Demographics

**Age (years) – Mean = 44.1 (Range = 27–69)**		
	20-30	62 (44.3%)
	31-59	57 (40.7%)
	60′s	19 (13.6%)
	Unspecified	2 (1.4%)
Gender		
	Female	85 (60.7%)
	Male	54 (38.6%)
	Unspecified	1 (0.7%)
Practice location (province)		
	AB	13 (9.3%)
	BC	89 (63.6%)
	NB	2 (1.4%)
	MB	17 (12.1%)
	ON	8 (5.7%)
	PE	1 (0.7%)
	SK	8 (5.7%)
	Unspecified	2 (1.4%)
Years working with PCa patients		
	0-5	63 (45%)
	6-20	38 (27.1%)
	21+	37 (26.4%)
	Unspecified	2 (1.4%)
Linkages to PCSGs		
	Presenter at group	9 (6.4%)
	Refereed patients to group	51 (36.4%)
	Group member	1 (0.7%)
	No linkages	79 (56.4%)

n.b. percentages may not total 100 due to rounding.

### Features of PCSGs that positively influence men’s adjustment to PCa

**Figure 1:**
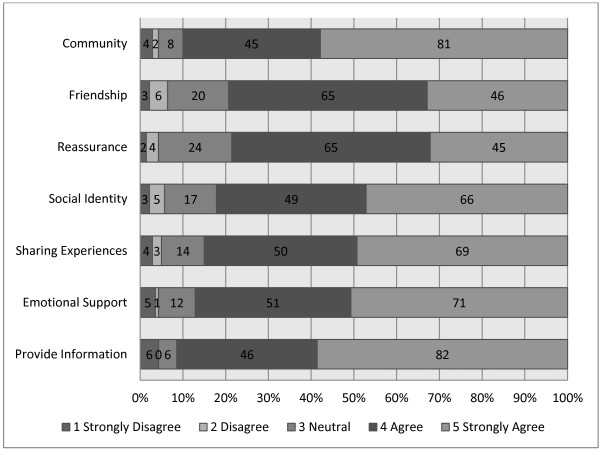
Positive factors how PCSGs influence men’s adjustment to PCa.

Respondents provided ratings of seven features of PCSGs that were potentially positive influences on men’s adjustment to PCa (Figure 1). The ratings of these features were uniformly high. Access to information and community support were identified as the biggest benefits of attendance, whilst friendship and reassurance were the least strongly endorsed. Yet this finding is relative, as 80% of the respondents agreed or strongly agreed that these latter features also had a positive effect on men’s adjustment to PCa, indicating they were still perceived as beneficial.

### Features of PCSGs that negatively influence men’s adjustment to PCa

PPs rated nine items related to possible negative influences of PCSGs including, meeting with dominant members who push their own views, dissemination of inaccurate information, hearing negative experiences, creating conflict over treatment decisions, promoting a specific clinician, creating confusion, supplying irrelevant information, causing confrontation and promoting a single therapy. The median response for all of these items was 3 (neutral) with no specific negative factors being singled out as particularly influential. Nevertheless, some negative perceptions of the value of PCSGs did arise in the open-ended responses, and are discussed below.

### Reasons for Attending PCSGs

**Figure 2:**
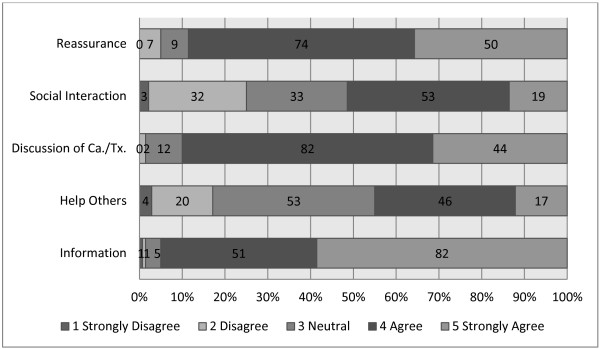
Reasons why men choose to attend a PCSG.

PPs rated five items in the survey related to why men attend PCSGs. The most highly endorsed reason to attend PCSGs was to gain information (median of 5), followed by discussion of PCa and therapies, and reassurance (median of 4; see Figure 2). This appears to be consistent with the findings concerning positive influences of PCSGs, where groups act as information trading resources and this feature is a primary motivator for attendance. There appeared to be less agreement on the value of social interaction and helping others to help promote PCSG attendance.

### Reasons for not attending PCSGs

**Figure 3:**
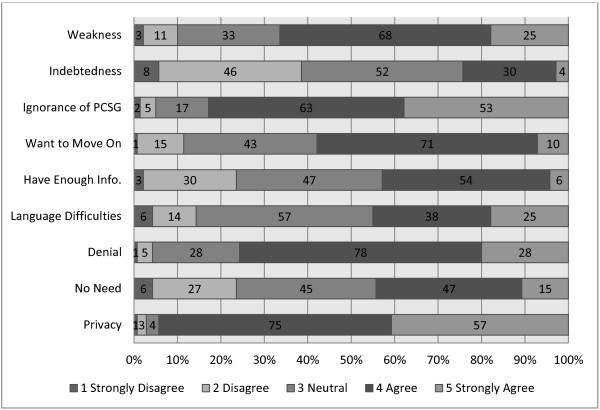
Reasons why men choose not to attend a PCSG.

Perceived reasons for not attending PCSGs were assessed with 9 items in the survey (see Figure 3). Privacy and ignorance of what PCSGs could offer were the most clearly endorsed reasons for not attending PCSGs (median of 4), illustrating concerns that men don’t want to discuss their problems with others, and also a perceived lack of patient’s knowledge about the groups. Denial, a desire to move past the PCa experience, a concern they may feel “indebted” and a perceived weakness in sharing emotion were also highly endorsed by PPs (median of 4) as reasons why some men might not want to attend PCSGs. Several respondents in the open-ended responses also identified language as a potential barrier to attendance.

### Characteristics of effective PCSGs

**Figure 4:**
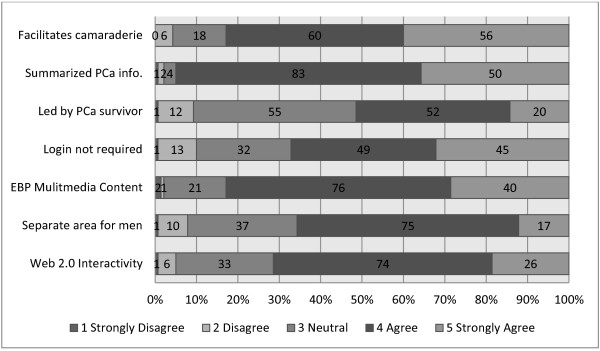
Reasons why men choose not to attend a PCSG.

Respondents were asked to rate six items designed to assess their views on the characteristics of effective face-to-face PCSGs (see Figure 4). Avoiding bias by not promoting one view of treatment was the most highly endorsed characteristic of effective PCSGs (median rating of 5). Having a trained facilitator, discussion of a diversity of therapies, having a range of different health profession’s input, getting support from other health organizations, and being patient driven were also generally agreed as the most important characteristics for group success (median rating of 4).

### Characteristics of Effective Web-based PCSGs

**Figure 5:**
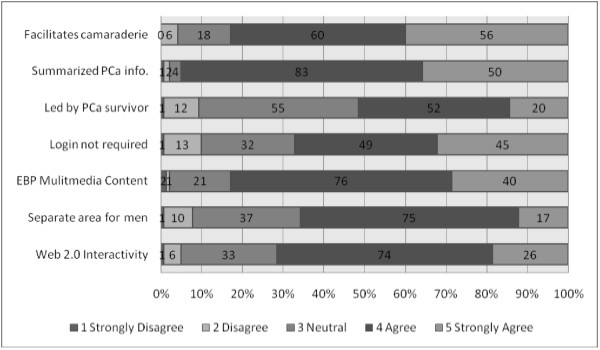
Key factors for the effectiveness of a web-based PCSG.

Subjects were also asked to consider a range of aspects of Web-based PCSGs, and consider what they thought would be most effective. They rated the seven items in this question positively (median of 4; see Figure 5). Provision of summarized PCa information was identified as the most essential feature of effective web-based PCSGs, closely followed by the provision of multimedia evidence-based HCP presentations. Facilitation fostering camaraderie was also highly valued, as was Web 2.0 interactivity. Over 60% thought that a separate web area for men would be important and a login should not be required. More mixed views with no consensus were evident as to whether the website should be led by a PCa survivor.

### Content-analysis of open-ended responses

Sixty-three of the respondents shared additional insights via written comments. Table 2 presents the five broad themes along with illustrative quotes derived from the content analysis of the open-text comments.

**Table 2:** Key thematic elements from open text questions (numbers indicate additional similar comments)

**Broad themes**	**Positive comments**	**Negative comments**
Marketing	*“Schedule groups during sporting event” “Need better advertising, pts don’t know where to go. MD’s don’t know where to refer. (3)*	*“I have never recommended or been aware of such groups.”*
		*“Please expand your services to other commonly used languages in Canada”*
	*There should be better promotion of prostate cancer support groups in order to “destigmatize” the challenges associated with the illness and its treatments.*	*“Should promote general men’s health, not just prostate cancer issues.”*
		*“…calling it a “support group” might scare away some men.”*
	*“Regularly scheduled.” (2)*	
	*“…media supported by government agencies.”*	
Inclusivity	*“Important to consider spouses experience with prostate cancer.” (3)*	*“…language + culture barriers are significant.” (4)*
	*“Ethnic diverse prostate support groups would be helpful.” (3)*	
Privacy	*“..an outlet for men to share their experience and stay anonymous may help other patients.” (2)*	*“The vast majority of men prefer not to talk in group setting” (2)*
Accessibility	*“Web based support groups are important.” (4)*	*“Elderly patients… are not comfortable with web-based services” (2)*
	*“There should be a support group in each community - if not web-based access available” (2)*	*“A good portion of the largest group are not on the web so an office based group program needed” (2)*
		*“…web based access important but how do we access/educate them [blue-collar workers).”*
Balance	*“I am strongly in favor of support groups that are led/driven by patient needs and interests.”*	*“As a physician…I was very reluctant to join any groups, fearing I would become the ’2nd opinion.”*
	*“The support group needs to be balanced to help patients work through this in a way that doesn’t alienate them.”*	*“I do not think have a group lead that’s a “survivor” is necessarily a good thing.”*
Education	*“…education is most important…” (2) “…dissemination of up-to-date treatment options.”*	*-*

The effective marketing of PCSGs was a significant theme as several respondents commented on the need for this. Many subjects mentioned the importance of increasing professional and public awareness of PCa and PCSGs to reduce stigma and break with male stereotypes. One PP noted that “…patients don’t know where to go and MDs don’t know where to refer.” Specific suggestions included having PCa and PCSGs-related pamphlets and written resources, or posters in multiple languages in PP offices. One PP confessed that he had had never recommended PCSGs to PCa patients, because he was not aware of the existence of PCSGs.

The need for inclusivity in PCSGs, particularly for PCSGs that cater to specific ethnic groups and partners/caregivers, was also highlighted. Several respondents suggested that partners and caregivers should be encouraged to attend and invited to ask questions and share their experiences. In contrast, privacy was also a significant concern for PCSG effectiveness. Several respondents recognized men’s reluctance to engage health services and highlighted the potential for web-based PCSGs to help overcome men’s concerns about confidentiality and anonymity (particularly in rural communities).

Accessibility to PCSGs in local communities and the need for web-based PCSGs to reach specific sub-groups including young, blue-collar, and rural based PCa patients with limited access to healthcare were highlighted. Several respondents expressed concerns about the elderly and their limited access to the Internet. There were also concerns that older PCa patients have limited computer literacy and/or access to the Web (see Table 2).

In agreement with the Likert responses, respondents saw a balanced range of health professionals’ input as a key factor and that patient-centred PCSGs were key to success. However, this did not necessarily translate to seeing benefits in having PCSGs actually led by PCa survivors, as some commented they thought this might not necessarily be productive.

Respondents also agreed that PCSGs educate PCa patients and raise awareness on a range of important topics including prognosis, up-to-date treatment options, and side effects.

## Discussion

In terms of methodological design, the study had a typically low response rate of approximately 10% for remotely administered survey questionnaires [31] and this may be improved through targeted communication strategies such as additional warning and follow up e-mails prior to and after distributing the survey [32]. The survey tool proved satisfactory in providing useful data to explore PPs perceptions and the open-ended questions generated useful supplementary data, supporting the use of mixed method data collection strategies. This indicates that the use of supplementary interviews may also be useful in future studies. No other specific technical or cultural issues arose from the methodology.

The pilot study indicates that information seeking is one of the main reasons men attend PCSGs (Figure 1 and Table 2), in supporting the findings of previously reported work [10,12,13]. The PPs recognized that many men do not want to self-disclose and have concerns that PCSGs might not meet their needs (Table 2), and these perceptions are also in line with previous research about why men don’t attend [10,12]. Language, ethnic and cultural barriers were all identified by PPs as barriers to PCSG effectiveness, suggesting that ensuring PCSGs are inclusive (including providing support for partners) may be important to encourage participation by diverse groups of men with PCa as well as garnering PPs referrals to PCSGs.

The views expressed (Figures 1, 2 & Table 2) indicated that PPs considered PCSG attendees would be less engaged with sharing in the groups, and more likely to attend in order to obtain information. In some respects, this may indicate that PPs do not anticipate men as longer-term attendees to these groups. The improved uptake of PCSGs would also seem contingent on improved strategic marketing as ignorance of PCSGs remains a concern (Figure 3 & Table 2). Formal linkages with government agencies, other community-based organizations and multidisciplinary HCPs could help support this. PPs’ core work involves providing information, and by encouraging PPs to more explicitly promote the availability and potential benefits of PCSGs, patients might be more likely to attend.

The respondents held positive attitudes toward Web-based PCSGs and endorsed their use as a useful step towards some men eventually participating in face-to-face group meetings or workshops (Figure 5 & Table 2). Although there were concerns that older PCa patients have limited computer literacy and/or access (Table 2), recent reports identify older adults as the fastest growing group of Internet and computer users [33] so this may not be such a serious issue as envisaged. The optimal structure and content for web-based PCSGs remain to be established, but other patient populations have effectively used Web 2.0 social media to share information, gain peer support, and even create a longitudinal database of disease progression, symptoms, responses to therapies and coping mechanisms (e.g., http://crohnology.com for Crohn’s and colitis patients).

There was no clear consensus amongst PPs on who was best placed to facilitate PCSGs. There was not a strong endorsement for PCa survivors to take on this role amongst the PPs in our sample (Figure 5). Although, overall being patient-driven PCSGs were seen as important (Figure 4), concerns about peer-led groups were also evident. While HCPs are likely to be well prepared to facilitate PCSGs, the involvement of HCPs in facilitating PCSGs has been a topic of concern in the past [34-36]. It may also be unrealistic to expect that HCPs can assume or sustain facilitation roles. Many lack knowledge about how the groups work [29,30,37] and they may not be able to take on additional responsibilities because of other demands on their resources [38]. Also there may be some overlap between PCSG psychosocial oncology services and those provided by PPs and other HCPs [39].

### Limitations

As an inductive exploratory pilot study with a population drawn from a convenience sample predominately made up of conference attendees, we do not know if the findings are generalizable to other PPs practicing within or outside Canada. The web-based survey approach also has limitations in terms of technological issues (lack of technological familiarity on the part of respondents or their willingness to use a computer to complete the survey, and browser incompatibility problems). That only 36% of the participants had referred patients to PCSGs, whilst 56% had no formal linkages to PCSGs can also be considered a limitation of this initial work. Nevertheless, no specific problems arose with the survey methodology or content and amid these limitations this foundational work indicates that PPs may need to be encouraged to raise awareness about PCSGs among PCa patients.

## Conclusions

These pilot study findings indicate that the approach and Steginga survey instrument used can be usefully applied in wider studies to explore PPs attitudes towards PCSGs, with careful attention to strategies to maximize response rates. Formal interviews to ascertain more detailed qualitative data to triangulate findings are also desirable. PPs value the role and potential of PCSGs to play an important educational role for PCa patients and their families. The PPs perceptions of PCSGs are positive and attitudes identified in this pilot study appear supportive of PP referrals to PCSGs. That said, if men and their partners are to benefit from PCSGs it is clear that PPs should be further encouraged to formally connect and endorse PCSGs as a legitimate resource for the psychosocial support of men who experience PCa and their families.

Identifying and addressing the perceived barriers to attending PCSGs, and strategic marketing initiatives are also important. PPs attitudes toward Web-based PCSGs suggest that this approach holds good potential for enhancing access among men who would not ordinarily attend a face-to-face group session. This initial work has provided some valuable insights into these issues and set the ground for larger–scale studies to explore these aspects in the wider Canadian context.

## Abbreviations

HCP: Health care provider; PP: Primary practitioners (aka General Practitioners); PCa: Prostate cancer; PCSGs: Prostate cancer support groups.

## Competing interests

The authors declare that they have no competing interests.

## Authors’ contributions

The study presented here was carried out in collaboration between all authors. JLO, JLB and MM contributed substantially to the conception and design of the study. JLO, JB, MM, BG and CSH carried out the participant recruitment and data collection. JLB, JSO, JLO and JB were involved in the data interpretation. BG, JLO and CSH drafted the initial manuscript, and JB, MM and JSO were involved in revising and providing substantial content and revisiting data analysis in the later phase of the manuscript production. All authors read and approved the final manuscript.

## Authors’ information

Dr. Bernard Garrett and Dr. John L. Oliffe are Associate Professors at the University of British Columbia, School of Nursing. Dr. Joan L. Bottorff is a Professor in the School of Nursing and Director of the Institute for Healthy Living at the University of British Columbia’s Okanagan campus. Dr. Michael McKenzie is a Radiation Oncologist at the BC Cancer Agency and Clinical Professor of the Division of Radiation Oncology and Developmental Radiotherapeutics at the University of British Columbia. Christina Han is a social science researcher at the University of British Columbia, School of Nursing. Dr. Ogrodniczuk is Professor of Psychiatry at the University of British Columbia.

## Pre-publication history

The pre-publication history for this paper can be accessed here:

http://www.biomedcentral.com/1471-2296/15/56/prepub
